# Structural conservation of WEE1 and its role in cell cycle regulation in plants

**DOI:** 10.1038/s41598-021-03268-x

**Published:** 2021-12-13

**Authors:** A. Détain, D. Redecker, N. Leborgne-Castel, S. Ochatt

**Affiliations:** 1 Agroécologie, AgroSup Dijon, INRAE, Univ. Bourgogne Franche-Comté, 21000 Dijon, France; 2 Agroécologie, AgroSup Dijon, CNRS, INRAE, Univ. Bourgogne Franche-Comté, 21000 Dijon, France

**Keywords:** Biotechnology, Cell biology, Developmental biology, Genetics, Molecular biology, Plant sciences

## Abstract

The WEE1 kinase is ubiquitous in plant development and negatively regulates the cell cycle through phosphorylations. However, analogies with the control of the human cell cycle by tyrosine- (Tyr-) phosphorylation of cyclin-dependent kinases (CDKs) are sometimes questioned. In this in silico study, we assessed the structural conservation of the WEE1 protein in the plant kingdom with a particular focus on agronomically valuable plants, the legume crops. We analyzed the phylogenetic distribution of amino-acid sequences among a large number of plants by Bayesian analysis that highlighted the general conservation of WEE1 proteins. A detailed sequence analysis confirmed the catalytic potential of WEE1 proteins in plants. However, some substitutions of an arginine and a glutamate at the entrance of the catalytic pocket, illustrated by 3D structure predictions, challenged the specificity of this protein toward the substrate and Tyr-phosphorylation compared to the human WEE1. The structural differences, which could be responsible for the loss of specificity between human and plants, are highlighted and suggest the involvement of plant WEE1 in more cell regulation processes.

## Introduction

The function of WEE1 was first described for yeast and then human as an inhibitory Tyr-phosphorylation of CDKs, leading to cell cycle arrest^1,2^. Later, plant WEE1-like proteins were repeatedly described as protein kinases that negatively impact plant cell division and growth, suggesting a conserved phosphorylation cascade between eukaryotes for cell cycle regulation^3–8^. Indeed, the phosphorylation of tyrosine residue(s) of CDKA;1 by plant WEE1s leads to a decrease in CDKA;1 activity^3,5,9,10^, similarly to human CDK1 and the fission yeast ortholog CDC2^1,2^. However, while repressing *WEE1* expression was shown to alter the development of tomato^5^, in *Arabidopsis thaliana*, *wee1* mutants were not morphologically different from the wild-type^10^. These data suggest a species-dependent importance of WEE1 in development regulation processes, and might consequently question the conservation of WEE1 in plants.

On the other hand, although the mechanisms of cell cycle regulation by WEE1 are still not completely clear, this protein remains strongly involved in stress responses. In this context, *WEE1* is highly expressed in *A. thaliana* in response to DNA damages or replication stress, to which *wee1* mutants are sensible^6,10^. These stress responses are controlled by the kinases ATM (Ataxia-Telangiectasia Mutated) and ATR (Rad3-related) from DNA repair signaling cascades^11^. The involvement of WEE1 in DNA repair cascades was recently confirmed with the inhibiting serine-phosphorylation of two new targets involved in cell cycle progression, PRL1^12^ and FBL17^13^. Furthermore, *WEE1* was shown to be overexpressed under drought and high salinity in *Medicago truncatula*^14,15^, and under salinity in *Brachypodium distachyon*^16^. Therefore, *WEE1* would be a potential candidate for functional studies on abiotic stress resistance aimed at developing more resilient genotypes for a sustainable agriculture under environmental constraints. Hence, the modulation of *WEE1* expression *in planta* will target new crops with agricultural value such as the legumes. To this aim, there is a need to analyze the conservation of this protein among plants of interest and to decipher possible differences that might affect the function and specificity between species as hinted above.

**Figure 1:**
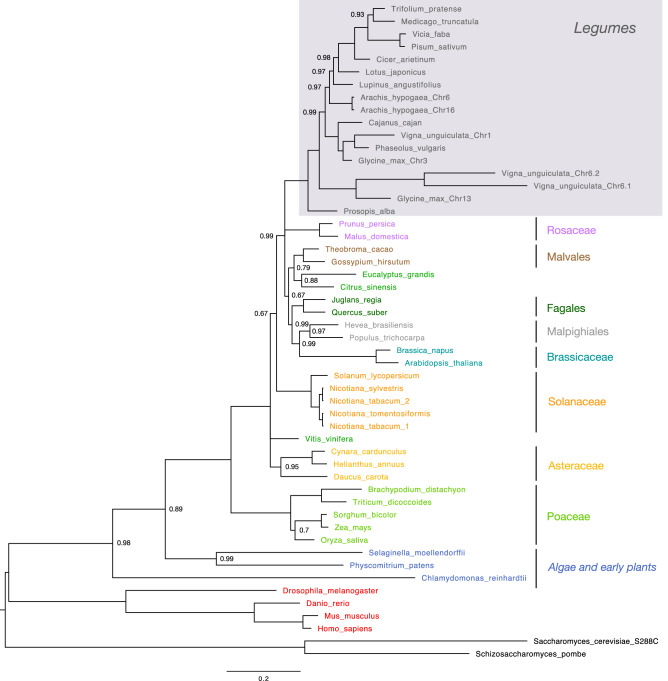
Evolutionary relationships among amino acid sequences of WEE1. The evolutionary history was inferred by Bayesian analysis using the program MrBayes. Node probabilities below 1 are indicated. The tree was rooted by midpoint rooting. Taxonomically-related organisms are indicated by the same color code, except for *V. vinifera*, *C. sinensis* and *E. grandis*. Legumes highlighted by the gray box are detailed in Fig. 2. Scale bar indicates number of expected changes per site.

**Figure 2:**
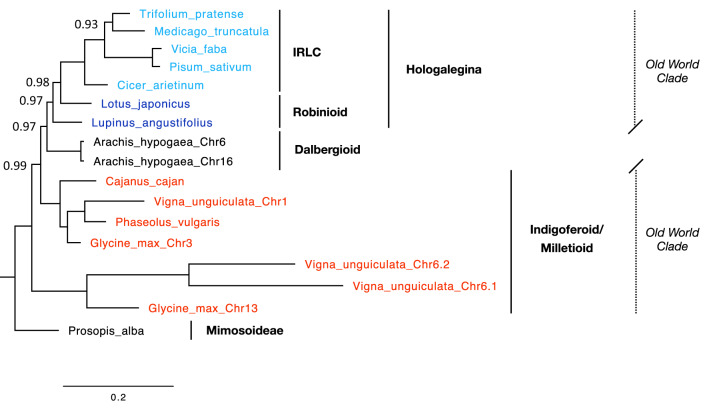
Detail of evolutionary relationships among amino acid sequences of WEE1 in Legumes. The evolutionary history was inferred by Bayesian analysis using the program MrBayes. Taxonomic groups are color-coded: Mimosoideae and Dalbergioid clade in black, Indigoferoid/Milletioid clade in red, Hologalegina in blue. Hologalegina are divided into Robinoid clade (dark blue) and IRLC (Inverted Repeat-Lacking Clade) (light blue). Trifolieae (*T. pratense* and *M. truncatula*) as well as Fabeae (*V. faba* and *P. sativum*) are also grouped. Node probabilities below 1 are indicated. Scale bar indicates number of expected changes per site.

**Figure 3:**
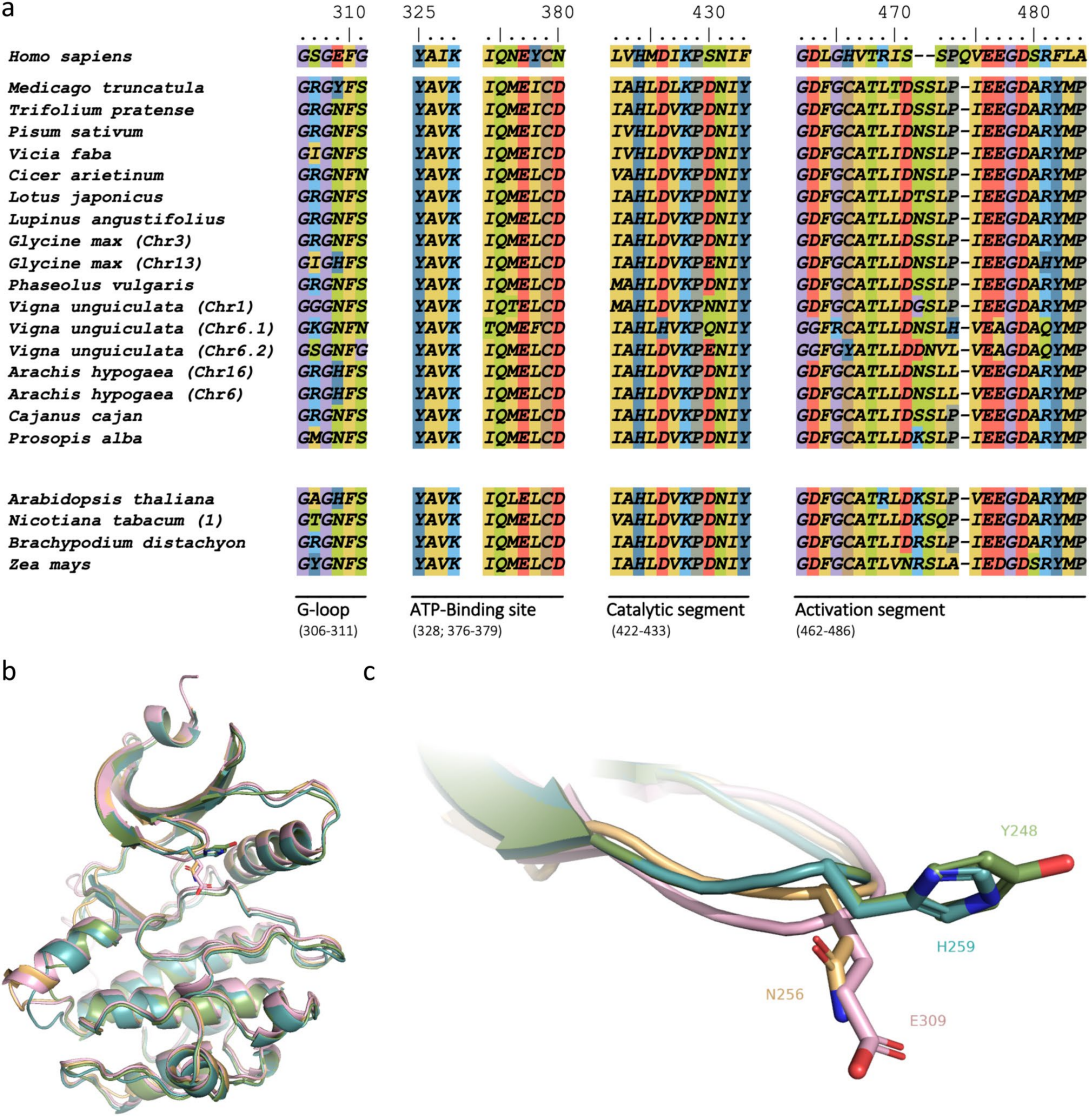
Sequence alignment of four segments of WEE1 for plants of interest and 3D predictions. (**a**) Sequences were aligned on MEGAX based on CLUSTAL W algorithm^29^. Alignments were then edited using BioEdit 7.2.5 software^31^ (Ibis Biosciences, CA). Here, only four segments are shown: G-loop, ATP-Binding site, Catalytic segment and Activation segment. Amino acid numeration is based on the human (*H. sapiens*) sequence. Amino acids are colored according to their properties: hydrophobic (AILMFVW) in yellow; acidic (DE) in red; basic (RK) in light blue; polar (QSNT) in green; other aromatic (YH) in dark blue; and C in brown, P in grey and G in purple. Legumes (middle) and model (/examples of dicot and monocot) plants (bottom) are shown. (**b**) Superimposition of the 3D structure of *H. sapiens* WEE1 (pink) with predicted ones of *A. thaliana* (blue), *M. truncatula* (green) and *P. sativum* (gold). (**c**) Detail of the 3D G-loops with the human E309 or its analogs (H259 in Arabidopsis, Y248 in *M. truncatula*, or N256 in *P. sativum*) with side chains represented in sticks.

In order to illustrate the conservation of plant WEE1 proteins, the phylogram presented in Fig. 1 depicts the evolutionary relationships among amino acid sequences from different plants and outgroup taxa. This phylogenetic tree of WEE1 obtained by Bayesian analysis corresponds largely to the consensus phylogeny established from other genes^17^, including the monocots/dicots separation and the position of the legume family reflecting a high degree of conservation of WEE1 across many plant lineages. Interestingly, long branches in the Brassicaceae coincide with amino-acid exchanges in the functional domain (see below), putting into question the adoption of *A. thaliana* as a model system to study this gene. The clear provenance of the two different versions of *WEE1* gene in *Nicotiana tabacum* (tobacco) from each of its parents, *N. sylvestris* and *N. tomentosiformis*, is noteworthy. Within the legume family (Fig. 2), WEE1 phylogeny recovers the well-characterized monophyletic group of Hologalegina (“temperate herbaceous tribes”)^18^, including the IRLC (Inverted Repeat-Lacking Clade)^19^. On the other hand, the Indigoferoid/Milletioid clade^19^, represented here by *Cajanus, Vigna, Glycine* and *Phaseolus*, which is known to be a sister group to the Hologalegina, forms a paraphyletic grade in the WEE1 phylogeny. Moreover, in known consensus phylogenies based on other genes, *Arachis*, belonging to Dalbergioid group, is outside the two mentioned groupings (“Old World Clade”)^19^. Multiple copies of WEE1 found in soybean (*Glycine max*) and cowpea (*Vigna unguiculata)* are highly divergent, with different copies not grouping by species. The copy on chromosome 13 of soybean and the two copies on chromosome 6 (Chr6.1 and Chr6.2) in cowpea might derive from a common duplication event. The substantial divergence may indicate gene duplication early in the evolution of legumes. Very long branches indicating a high substitution rate could be hints for a change of gene function as also suggested by amino acid exchanges in the catalytic domain and other functionally essential regions (Fig. 3a). Duplication of WEE1 genes is not restricted to legumes and plants but has already been documented in metazoans and fungi^20^. Using only two plant sequences, Sorrel et al.^4^ found that the catalytic domain was conserved across animals and plants and that the two plants sequences grouped together. Here, we provide a much more comprehensive phylogenetic analysis based on 46 plant and algal sequences, and we show that WEE1 sequences follow the expected phylogeny. This highlights the kinase domain conservation of our protein of interest between plants and animals.

A more detailed analysis of the WEE1 sequences was therefore necessary to better decipher the conservation of this protein within the plant kingdom. To address this, we used the well-described human (*Homo sapiens*) sequence that has been characterized by X-ray crystallography and already reviewed in detail^21,22^. Thus, we focused on four amino acid segments from the kinase domain that are essential for the catalytic activity and specificity (Fig. 3a; Supplementary Fig. S1). In this respect, the catalytic segment is well-conserved among all plant sequences studied here, except for *V. unguiculata* Chr6.1 that lacks the essential catalytic aspartate (D) residue located at position 426 in human. For the remaining sequences, catalytic segments match the kinase consensus sequence IVHxDLKPxNIx already described^21^, with some minor differences for hydrophobic amino acids (Fig. 3a, in yellow). In the activation segment, the signatory “EGD” motif (477–479 in human)^4^ is well-conserved except in two different cases. First, for *Sorghum bicolor*, *Zea mays*, *Oryza sativa* and *Selaginella moellendorffii* the glutamate (E) residue is replaced by an aspartate (D) residue. Secondly, in both WEE1 versions of *V. unguiculata* encoded on chromosome 6 the “E” is replaced by a hydrophobic small alanine (A) residue. In addition, for these two last sequences, the aspartate (D) residue is replaced by a glycine (G) residue in the “DFG” (463–465) motif of the activation further upstream in the sequence. This highly conserved aspartate of the DFG motif, known as D463 in the DLG human WEE1, is essential for the Mg^2+^-ATP binding. Therefore, we can safely assume that the *WEE1* duplicated genes in *V. unguiculata* might not encode for functional WEE1 protein, especially for the “Chr6.1” sequence, which lacks both catalytic and ATP-binding aspartates. With this latter exception, the activation segment that maintains the protein in an active state is well-conserved among plants. However, the phenylalanine (F) residue of the DFG motif, which is present in all plants and Ascomycetes, is also present in human Myt1 protein (belonging to WEE family), whilst in animal WEE1 it harbors a leucine (L464)^22^. Moreover, the following residues that interact directly or indirectly with ATP are conserved between human and plant WEE1 sequences, namely C379, K328, K428, N431, D463^21^.

**Figure 4:**
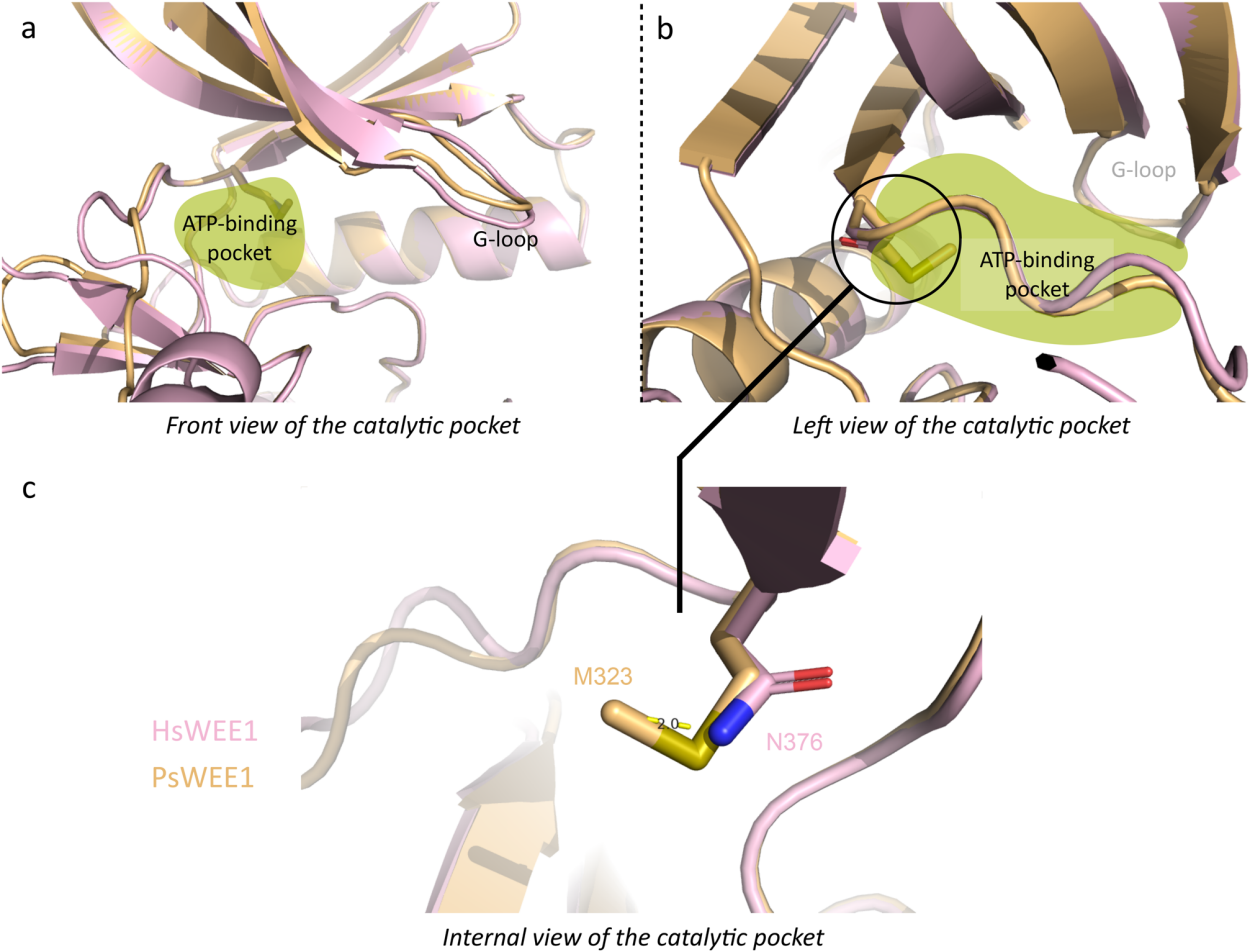
ATP-binding pocket and steric hindrance of human N376. (**a**) Front and (**b**) left views of the *H. sapiens* WEE1 (pink) 3D structure superimposed on *P. sativum* (gold) predictions in cartoon representation. The ATP-binding pocket is highlighted in green. (**c**) Internal view of the catalytic pocket with *Hs*N376 and *Ps*M323 represented in sticks. The distance (yellow hatched lines) of 2 Å between the two residue extremities is shown.

Interestingly, one amino acid is not conserved between plants and animals in the ATP-binding pocket. This is the asparagine (N) residue at position 376 in human WEE1, which is responsible for the “gate-keeper” effect described in comparison to human Myt1 that presents a threonine at this position^22^. This difference between Myt1 and WEE1 in human could be one reason for inhibitor affinity and specificity^22^. Indeed, in human WEE1 the loop including residues 376–379 forms a hinge region at the back of the ATP-binding pocket, and residues interact partially with the ATP adenine or its inhibitors^21,22^. For most plant WEE1 proteins, a methionine (M) residue occupies the position (Fig. 3a), with a side chain 2 Å longer than an asparagine residue (Fig. 4). The replacement by a methionine induces a residue change in size and polarity that should be considered for the eventual use of WEE1 specific inhibitors in plants.

The capacity of WEE1 to target tyrosine residues on CDK proteins was previously described in plants^9,10^. The role of Tyr-phosphorylation of CDKA;1 in cell cycle regulation was debated^23^, and the capacity of WEE1 to phosphorylate other residue and protein types was recently proven^12,13^, whereby WEE1 specificity in plants might differ compared to human. Therefore, we further characterized other features that could influence the specificity of WEE1 toward the target. First, both the aspartate 479 (D479) and arginine 481 (R481) in human are fully conserved among plants, except for the arginine replacement in both *V. unguiculata* Chr6.1 and 6.2, and *G. max* Chr13 (Fig. 3a). R481 residue holds D479 in a position playing a key role in substrate recognition and binding through a double hydrogen-bonded salt bridge^21^. However, in human the target peptide inserts itself over two arginine side chains, R481 and R518, for which only R481 is conserved amongst plants, while R518 found in human seems to only be conserved in mice (*Mus musculus*). Thus, residue changes at this position could lead to alternate substrate binding. In plants, the second arginine residue (R518) is mostly replaced by a far less bulky glutamate (E) residue, leading to a less restricted access to the binding site (Supplementary Fig. S2).

**Figure 5:**
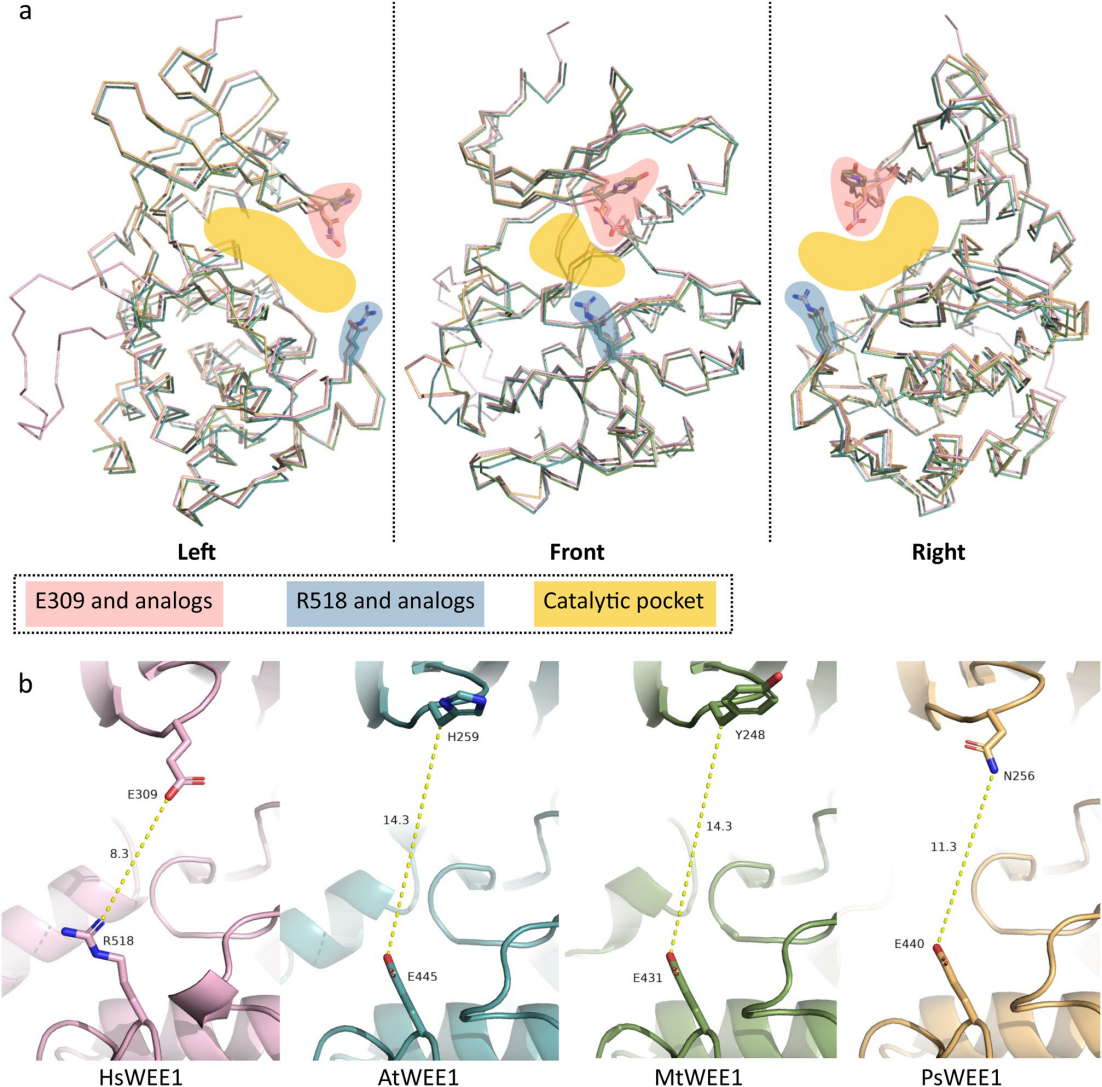
Access to the catalytic pocket of WEE1. (**a**) Front and side views of *H. sapiens* WEE1 (pink) 3D structure superimposed to *A. thaliana* (blue), *M. truncatula* (green) and *P. sativum* (gold) predictions in ribbon representation. Residues conditioning the access to the catalytic pocket are highlighted in pink for E309 and its analogs and in blue for R518 and its analogs. The resulting catalytic pocket space approximately occupied by the target is highlighted in yellow. (**b**) Distance measurement (yellow hatched lines) in Å between residues conditioning the access to the catalytic pocket for the four 3D structures.

In addition to the two arginine residues on which the target peptide is docked, the catalytic pocket contains a glutamate residue above, located in the glycine-rich loop (G-loop) at position 309 in human WEE1^21^. This E309 was defined as responsible for the specific phosphorylation of tyrosines. This is supported by comparison with the close WEE protein Myt1, which has a smaller residue (namely a serine at position 120) allowing phosphorylation of both threonine and tyrosine residues^22^. In the case of human, the approach of the phosphorylation site of CDK1 is limited by E309 of WEE1 preventing any action on threonine 14, which is shorter than tyrosine 15^21,24^. Interestingly, plant WEE1s studied do not possess any glutamate nor serine residue at this position but a histidine (H), tyrosine (Y) or asparagine (N) instead, as shown for *A. thaliana, M. truncatula and Pisum sativum*, respectively (Fig. 3a). These three possibilities are illustrated with three WEE1 3D predictions in these plants (Fig. 3b,c; Supplementary Video S1). Compared to human WEE1, steric hindrance appears to differ between the four structures. Glutamate and asparagine side chains expose the same orientation compared to histidine and tyrosine. In addition, asparagine and histidine residues are respectively less bulky than glutamate and tyrosine. Taken together, these data describe small differences in plant WEE1s that can affect substrate access to the catalytic pocket. In addition, distance measurement between the R518 and E309 in human reveals an access of 8.3 Å (Fig. 5); whereas the three 3D predictions of plant sequences show a larger access, of 14.3 Å for *A. thaliana* and *M. truncatula*, and 11.3 Å for *P. sativum*.

WEE1-like proteins are deeply involved in plant development and are hence genetically present in at least one copy for all plants studied here. Despite a Tyr-phosphorylation role demonstrated for human WEE1, this protein is closer to Ser/Thr kinase families^25^. The data depicted in Fig. 1 shows that WEE1 is in general well-conserved across the plant kingdom and close to animal proteins in agreement with results by Sorrell et al. comparing animals with *A. thaliana* and *Zea mays*^4^. The catalytic potential in plants has been maintained during evolution with conservation of the consensus kinase sequence, harboring the essential catalytic aspartate and an ATP-binding capacity. However, slight modifications in the back ATP-binding pocket, where the N376 found in human is not conserved, should be considered for the use of specific plant inhibitors. In addition, in this study we highlighted two other modifications compared to human WEE1 that could be related to target specificity, especially the non-conserved R518 and E309. Substitution of R518 by glutamate residues led to a less restricted access to the catalytic pocket. This could explain why it has recently been shown that WEE1 targets not only CDKA;1 but also other proteins such as PRL1^12^ or FBL17^13^ in *A. thaliana*. Since the role and target specificity of WEE1 has been questioned in plants^12,13,23^, it has been postulated that WEE1 can target other proteins and maybe phosphorylate other residues. Further, except for two recent publications demonstrating serine-phosphorylation by WEE1^12,13^, previous studies mostly focused on Tyr-phosphorylation events and not on threonine nor serine events that could miss out the substrate. In plants, the residue identified for Tyr-phosphorylation specificity is not conserved and WEE1 proteins are related to Ser/Thr kinases. We can therefore assume that during evolution in human the protein specialized toward the phosphorylation of tyrosine by acquiring a glutamate residue, which prevents catalytic action on shorter residues. However, in plants this non-conservation supports the idea of having other proteins and residues such as threonine or even serine targeted by WEE1. Our 3D predictions (Fig. 3b,c) show a substitution of the E309 by either a shorter asparagine, or by a histidine or tyrosine that seem not to have the same side chain orientation. Such substitution can result in deeper accessibility to the catalytic site by residues shorter than tyrosine (i.e. threonine or serine). Therefore, WEE1 in plants does possess molecular features to target other proteins and residues that should be considered for the understanding of its role. Hence, unlike in human, WEE1 could be involved in more plant developmental processes and especially in various stress responses^14–16,26,27^. Previous studies concurred in showing a conservation of the expression profiles of *WEE1* for plants subjected to a variety of stresses or under development processes including taxonomically distant species like *Solanum lycopersicum*^5^, *Zea mays*^3^, *A. thaliana*^4,6^, *M. truncatula*^14,15^ and *Brachypodium dystachion*^16^. Indeed, this is correlated to a transcript accumulation in replicating nuclei^3–6,28^. As with the structure, we also showed here a remarkable conservation of the gene function across species although slight differences could be observed among plants, especially between model and agronomically important plants such as legumes that will have to be considered in the development of novel stress resilient crops.

## Material and methods

### Data collection

Amino acid sequences of WEE1 were retrieved from NCBI based on tBlastn of *H. sapiens* WEE1A (NP_003381.1) and *M. truncatula* WEE1 (XP_003625897.1). From identified cDNA sequences, affiliated amino acid sequences were compared on CLUSTAL W to the translated nucleic sequence obtained using the ExPASy *translate tool* to check for correspondence.

Except for *P. sativum* (URGI-Versailles: Psat3g023800.1), *Lotus japonicus* (KEGG: Lj1g3v4913220.1) and *Vicia faba*^29^, all amino acid sequences used in this study can be found in NCBI (https://www.ncbi.nlm.nih.gov): *Glycine max* Chr3 (NP_001237183.2), *Glycine max* Chr13 (XP_028197085.1), *Phaseolus vulgaris* (XP_007163086.1), *Vigna unguiculata* Chr1 (XP_027919536.1), *Vigna unguiculata* Chr6.1 (XP_027932705.1), *Vigna unguiculata* Chr6.2 (XP_27933621.1), *Cajanus cajan* (XP_020212943), *Cicer arietinum* (XP_004494355.1), *Arachis hypogaea* Chr16 (XP_025663287.1), *Arachis hypogaea* Chr6 (XP_025604099.1), *Lupinus angustifolius* (XP_019418127), *Trifolium pratense* (PNX96768.1), *Prosopis alba* (XP_28775925.1), *A. thaliana* (NP_171796.1), *Brassica napus* (XP_022547230), *Nicotiana tabacum* 1 (NP_001311853.1), *Nicotiana tabacum* 2 (XP_016436283.1), *Nicotiana tomentosiformis* (XP_009627373.1), *Nicotiana sylvestris* (XP_009761648.1), *Solanum lycopersicum* (NP_001234875.1), *Oryza sativa* (XP_015626457.1), *Triticum dicoccoides* (XP_037445611.1), *Brachypodium distachyon* (XP_024318260.1), *Zea mays* (NP_001335045.1), *Sorghum bicolor* (XP_021314934.1), *Daucus carota* (XP_017237690.1), *Helianthus annuus* (XP_021968973), *Cynara cardunculus* (XP_024967733.1), *Selaginella moellendorffii* (XP_002969867.2), *Physcomitrium patens* (XP_024398331.1), *Populus trichocarpa* (XP_02444062.1), *Malus domestica* (XP_008366563.3), *Prunus persica* (XP_007201523.1), *Quercus suber* (XP_023892780.1), *Juglans regia* (XP_018835462.1), *Citrus sinensis* (XP_006479906.1), *Eucalyptus grandis* (XP_039154644.1), *Gossypium hirsutum* (XP_016710737.1), *Theobroma cacao* (XP_007050841.2), *Hevea brasiliensis* (XP_021675081.1), *Vitis vinifera* (XP_002268578.2), *Chlamydomonas reinhardtii* (XP_001702079.1), *Mus musculus* (NP_033542.2), *Danio rerio* (NP_001296364.1), *Drosophila melanogaster* (NP_477035.1), *Schizosaccharomyces pombe* (NP_587933.1) and *Saccharomyces cerevisiae* (NP_012348.1).

### Alignment and phylogeny

Sequences were first aligned using CLUSTAL W in MEGAX 10.1.8^30^ using default parameters. Ends, where some sequences were incomplete, were truncated to improve alignment quality. Alignments were then edited using BioEdit 7.2.5 software^31^ (Ibis Biosciences, CA). The consensus phylogram was calculated by Bayesian analysis using the MrBayes 3.2.7 program and a mixed model over one million generations, sampling trees every hundredth generation^32^. The average standard deviation of split frequencies was 0.003114 < α (= 0.01). The consensus phylogram was drawn using FigTree^33^.

### 3D structure prediction and visualization

Two plant models (*A. thaliana* and *M. truncatula*) and one agronomically interesting plant (*P. sativum*) were chosen for 3D prediction of WEE1, that allow the representation of the three main substitution possibilities in plants of glutamate 309 from the human sequence. The three predictions were designed with SWISS-MODEL using the human WEE1A as template of which the kinase domain was crystallographied (1 × 8b)^21^. Prediction parameters are *At*WEE1 (Seq Identity = 40.48%; QMEAN = − 1.55), *Mt*WEE1 (Seq Identity = 40.32%; QMEAN = − 2.07), *Ps*WEE1 (Seq Identity = 41.60%; QMEAN = − 2.48), and local quality estimates presented in Supplementary Fig. S3. Then, predictions were superimposed on the human 3D structure in PyMOL^34^, followed by analyses and measurements carried out with this program.

## Supplementary Information

Supplementary Information 1.

Supplementary Figures.

Supplementary Information 2.
