# Supplementary material for: Structural conservation of WEE1 and its role in cell cycle regulation in plants
Source: Sci Rep. 2021 Dec 13;11:23862. doi: 10.1038/s41598-021-03268-x (PMC8668995; doi:10.1038/s41598-021-03268-x)
Supplement: Supplementary file 1 — Supplementary Information 1. [file 41598_2021_3268_MOESM1_ESM.docx]

Supplementary figure 1 | **Sequence alignment of all species studied.** Sequences were aligned on MEGAX based on CLUSTAL W algorithm^29^. Alignments were then edited using BioEdit 7.2.5 software^31^ (Ibis Biosciences, CA). Four segments are indicated below their location: G-loop, ATP-Binding site, Catalytic segment and Activation segment. Amino acid numbering starts from the beginning of the alignment, amino acids are colored according to their properties: hydrophobic (AILMFVW) in yellow; acidic (DE) in red; basic (RK) in light blue; polar (QSNT) in green; other aromatic (YH) in dark blue; and C in brown, P in grey, and G in purple.

Supplementary figure 2 | **Arginine residues conditioning the access to the catalytic pocket.** (**a**) Catalytic pocket of *Homo sapiens* WEE1 (pink) 3D structure superimposed on *Arabidopsis thaliana* (blue), *Medicago truncatula* (green) and *Pisum sativum* (gold) predictions in cartoon representation (**b**) with a zoom on the two human arginine residues R481 and R518 and their analogs. (**c**) Front and (**d**) back views of the two *H. sapiens* and *P. sativum* arginine that present the lowest shift of 0.5 Å between the two alpha carbons of *Hs*R518 and *Ps*E440. The distance (yellow hatched lines) of 3.4 Å between residue extremities is shown.

Supplementary figure 3 | **Local quality estimate for the three predictions with SWISS-MODEL.** (**a**) *At*WEE1 (**b**) *Mt*WEE1 and (**c**) *Ps*WEE1.

Supplementary Video 1| **3D clip presenting the superimposition of the four WEE1 structures and zoom on the G-loop (Fig. 2).** *Homo sapiens* WEE1 (pink) 3D structure superimposed on *Arabidopsis thaliana* (blue), *Medicago truncatula* (green) and *Pisum sativum* (gold) predictions in cartoon representation.
