# Supplementary material for: Structural conservation of WEE1 and its role in cell cycle regulation in plants
Source: Sci Rep. 2021 Dec 13;11:23862. doi: 10.1038/s41598-021-03268-x (PMC8668995; doi:10.1038/s41598-021-03268-x)

Supplementary Figure 1

```
                                          10        20        30        40        50        60        70
                                 ....|....|....|....|....|....|....|....|....|....|....|....|....|....|
Medicago truncatula              NYVSHSAATLRCRAIPPPCFSNPYLKDLSQKET-DPYANQ--RSKCAGFFPKFTAY----DGLSRYRTDF
Trifolium pratense               NYVSHSAAALRCRAMPPPCISNPYLKDVSQKETKDPFANQ--RSKCAGLFPKFTAV----DGLSRYRTDF
Pisum sativum                    NYVSQSAAALRCRAMPPPCYRNPYLKDVSQKET-DPFANQ--RSKCAGFFPKFTAN----EGLSRYHTDF
Vicia faba                       NYVSQSAAALRCRAMPPPCYRNPYLKDVSQKET-DPFANQ--RSKCAGFFPKFTSD----EGLSRYHTDF
Cicer arietinum                  NYVSHSAAALRCRVMPPPCISNPYLKDVSQQET-DPFANQ--RSKCAGLFPNFTSA----DGLSRYRTDF
Lotus japonicus                  NYVSHSAVALRCRAMPPPCIRNPYLKDVSKMET-DPFGNQ--RSKCAGLFPGFGGG----DGLSRYRTDF
Lupinus angustifolius            NYVSHSAVALRCRVMPPPCISNPYLKEVSKKET-DPFGNQ--RSKCAGLFPPTIGG----DGLSRYHTEF
Glycine max (Chr3)               NYVSHSAVALRCRVMPPPCFRNPYLKDVSDKDL-DPFGNQ--RLKCAGLFPAFTGG----DGLSRYRADF
Glycine max (Chr13)              NYVSRSAVALRCRVMPPPCIRNPFLKCLSEKEI-DPFESQ--RSKFAGLFPALVGGGGGGDCLSRYRNDF
Phaseolus vulgaris               NYVSQSAVALRCRVMPPPCFRNPYLKDVSEKER-DPFGNQ--RLKCAGLFPANSG-----DGLSRYRADF
Vigna unguiculata (Chr1)         NYVSQSAIALRFGVMPPPCFRNPFLNNVTDEEI-DPFRNQ--RLKCAGLFPAISGG----DGIPRYRSEF
Vigna unguiculata (Chr6.1)       -HTSRSAG-----CRPPLCNRNSFLNSNSEDEE-NIFRIQTLKWNDYGNGPPSIIG---SNGSSRYINDF
Vigna unguiculata (Chr6.2)       RYRSLSAMALMRRVTPPACVTNPYLKNISAGKR-EPFIYQ--RSTSPGLCPSLVGG---RRGLSRYHNDF
Arachis hypogaea (Chr16)         NYVSHSAVALRCRVMPPPCIQNPYLKDVSQKET-DPFGNQ--RIKCAGLFPGITGD----DGLSRYRTDF
Arachis hypogaea (Chr6)          NYVSHSAVALRCRVMPPPCIQNPYLKDVSQKET-DPFGNQ--RIKCAGLFPGITGD----DGLSRYRTDF
Cajanus cajan                    NYVSHSAVTLRCRAMPPPCFRNPYLKDVSKTEI-DPFENQ--RLKCAGLFPAVTGG----DGLSRYRTDF
Prosopis alba                    NYLSHSVLALRSRVIPPPCIRNPYLKDGLDKDK-DPYGNQ--RSKYAGLFPAITGG----DGLSRYRTDF
Arabidopsis thaliana             GYVSQAAVALRCRAMPPPCLKNPYVLNQSETAT-DPFGHQ--RSKCASFLPVSTSG----DGLSRYLTDF
Brassica napus                   GYVSRSAVALRSRVMPPPCLKNPYVMNESDTAT-DPFGYQ--RSKCASFLPASMGG----DGLSRYLTDF
Nicotiana tabacum (1)            SYVSQSAIALRYRVMPPPCIRNPYLRDASEIDV-DPFGNR--RSKCAGFNPVIFGN----DGLSRYRSDF
Nicotiana tabacum (2)            SYVSQSAIALRYRVMPPPCIRNPYLRDASEIDV-DPFGNR--RSKCAGFNPVIFGN----DGLSRYRSDF
Nicotiana tomentosiformis        SYVSQSAIALRYRVMPPPCIRNPYLRDASEIDV-DPFGNR--RSKCAGFNPVIFGN----DGLSRYRSDF
Nicotiana sylvestris             SYVSQSAIALRYRVMPPPCIRNPYLRDASEIDV-DPFGNR--RSKCAGFNPVIFGN----DGLSRYRSDF
Solanum lycopersicum             SYVSQSAIALRYRVMPPPCIRNPYLGDASEIDA-DPFGNR--RSKYPGFNPAISGN----DGLSRYRTDF
Brachypodium distachyon          SIVPQSARTLRSQVTPPPCIKNPY-NADPRIDD-GVFNVR--QGKSSGSSPSIGAY-----GLSRYRSDF
Triticum dicoccoides             SYVPHSARVLRSQVTPPPCIKNPY-NMDPRIDD-NVFSVR--KCKSSGSSPSIGAD-----GLSRYRTDF
Oryza sativa                     SYVSQSAVALRCRVMPPPCIRNPYLNTDHQIDD-NVFGGR--QCKSSGFSPSVDGD-----GLSRYRTDF
Zea mays                         GYVSQSAVALRCRVMPPPCVKNPYLNTDPCIDA-AVYGGR--QCNSAVFSPSIGGN-----GLSRYRTDF
Sorghum bicolor                  GYVSQSAVALRCRVMPPPCVKNPYLNTDPCIDD-AVYGGR--QCNSAGFSPSIGGN-----GLSRYRTDF
Daucus carota                    SYVSQSAVALRCRVMPPPCMKNPYLMDDSGVDI-DPFGSS--RSKCEGLFPSSISG----DGLSRYRTDF
Helianthus annuus                GYVSQSAVALRCRVMPPPCMKNPYIKDASENDA-DPFGDR--RSKCAVFFPAALGG----DGLSRYRTDF
Cynara cardunculus               GYVSQSAVALRCRVMPPPCMRNPYIKDASENDI-DPFGDR--RSKCAVFLPVAFGG----DGLSRYRTDF
Populus trichocarpa              NYVSQSAVFLRCRVMPPPCIKNPYLTDASEVGI-DPFGNQ--RSKCAGFFAAFAGG----DGLSRYHTDF
Malus domestica                  NYVSQSAVALRCRVMPPPCLKNPYLKDASEIGI-DPFGNQ--RSK--AFFPAFMGG----NGLSRYCADF
Prunus persica                   NYVSQSAVALRCRVMPPPCFKNPYLKDASEMDI-DPLGNQ--RSKCAAFFPAIMGG----NGLSRYCADF
Quercus suber                    SYVPQSAVALRCRVMPPPCIKNPYLMGASEMDI-DPFGNQ--RSKCAGFFQGLIGG----DGLSRYHTDF
Juglans regia                    SYVSQSAVALRCRVMPPPCIKNPYLMGASEMDL-DPFGNR--RSKCAGFFPALTGG----DGLSRYHTDF
Vitis vinifera                   SYVSQSAVALRCRVMPPPCLKNPYLKNASETDI-DPFGNR--RSKYSGFFPALIGG----DGLSRYHTDF
Citrus sinensis                  NYVPQSAVALRCRVMPPPCIKNPYQKDASEMDI-DRFGNQ-- AKCAGLFPVITGG----DGLSRYYTDF
Eucalyptus grandis               SYVPQSAIALRCRVMPPPCIRNPYLSDALETDI-DPFGNQ--RSKCAGLFSSITGG----DGLSRYHTDF
Gossypium hirsutum               NYVSQSAVALRCRVMPPPCIKNPYLKDASEVDT-DPFGNQ--RSECAGFFPAIIGG----DGLSRYHTDF
Theobroma cacao                  NYVSQSAVALRCRVMPPPCIKNPYLKDASEMDT-DPLGNQ--RSKCAGFFPAIIGG----DGLSRYHTDF
Hevea brasiliensis               NYVSQSAVALRCRVMPPPCIKNPYLMDSSEVDV-DPYGNQ--RSKCAGFFSTIVGG----DGLSRYHTDF
Selaginella moellendorffii       -----KTLSLRQRVMSPPCFRNPFVNSDVFPER-----------ARQGCVSAFGSS-------SRYREEF
Physcomitrium patens             --------------QSPVSIQNPFLLEDTNLPQ------K--MITSAKITEDEWEQ----TFISIYRHDF
Chlamydomonas reinhardtii        KALSGRPPIPKLREVQPPMPRNPYLVRADDDNH-----------VFQASQSCLNRG-----NYSRFLWDY
Schizosaccharomyces pombe        FENTVSIHMDGRQPSPIKSRNNNQMSFAMEEEADVSQPSS--SSFTLSFPSALTSSKVSSS-TSHLLTRF
Saccharomyces cerevisiae S288C   RKSIIGATSQTHRESRPLSLSSAIVTNTTSAETHSISSTD--SSPLNSKRRLISSNKLSANPDSHLFEKF
Drosophila melanogaster          TQFGRENLNLNVNAMQKY-LLSDACDDDVTEEAGDSMREIHQQAP---KRLALHDT----NIS-RFKREF
Danio rerio                      NPFTPDSILVQSSTLQRNNRKRSHWNDSCGEDMDASDAEIEDELIPPSKRITMMEN----NMMSRYASEF
Mus musculus                     NPFTPDPVLLHSSGRCRG-RKRAYFNDSS-EDMEASDYEFEDETR-PAKRITITES----NMKSRYTTEF
Homo sapiens                     NPFTPDSLLLHSSGQCRR-RKRTYWNDSCGEDMEASDYELEDETR-PAKRITITES----NMKSRYTTEF
```

```
                                          80        90       100       110       120       130       140
                                 ....|....|....|....|....|....|....|....|....|....|....|....|....|....|
Medicago truncatula              HEIE-------------------------------QIGRGYFSCVFKVLKRIDG-CLYAVKRST-QFQLD
Trifolium pratense               HEIE-------------------------------QIGRGNFSCVFKVLKRIDG-CLYAVKRSTRQLQLE
Pisum sativum                    EELE-------------------------------QIGRGNFSCVFKVLKRIDG-CLYAVKRSTRQLHLE
Vicia faba                       EELE-------------------------------QIGIGNFSCVFKVLKRIDG-CLYAVKRSTRQLHLE
Cicer arietinum                  HEIE-------------------------------QIGRGNFNSVFKVLKRIDG-CLYAVKLSTRQLHQE
Lotus japonicus                  HEIE-------------------------------LIGRGNFSTVFKALKRIDG-CLYAVKHSTRQLHQE
Lupinus angustifolius            HEIE-------------------------------QIGRGNFSSVFKVLKRIDG-CLYAVKHSSRQLRLE
Glycine max (Chr3)               HEIE-------------------------------QIGRGNFSSVFKVLKRIDG-CLYAVKHSTRPLRLE
Glycine max (Chr13)              HEIE-------------------------------QIGIGHFSNVFKVIKRIDG-CLYAVKHSTRKLCKE
Phaseolus vulgaris               HEIE-------------------------------QIGRGNFSNVFKVLKRIDG-CLYAVKQSIRPLLLE
Vigna unguiculata (Chr1)         HEIE-------------------------------LIGGGNFSHVFKVLRRLDG-CLYAVKRSIRPLRLE
Vigna unguiculata (Chr6.1)       REIK-------------------------------EIGKGNFNKVFKVMNRLDG-CMYAVKRTIERLRTE
Vigna unguiculata (Chr6.2)       NEIE-------------------------------KIGSGNFGNVFKVLRRVDG-CLYAVKQTSDQLCKN
Arachis hypogaea (Chr16)         HEIE-------------------------------QIGRGHFSSVFKVLKRIDG-CLYAVKRSARQLRLE
Arachis hypogaea (Chr6)          HEIE-------------------------------QIGRGHFSSVFKVLKRIDG-CLYAVKRSARQLRLE
Cajanus cajan                    HEIQ-------------------------------QIGRGNFSSVFKVLKRIDG-CLYAVKHSTRQLRLE
Prosopis alba                    HEIE-------------------------------QIGMGNFSNVFKVLKRIDG-CLYAVKHSTRQLCLE
Arabidopsis thaliana             HEIR-------------------------------QIGAGHFSRVFKVLKRMDG-CLYAVKHSTRKLYLD
Brassica napus                   HEIQ-------------------------------QIGAGNFSRVFKVLKRIDG-CLYAVKHSTRKLYLD
```

```
Nicotiana tabacum (1)            HEIE-------------------------------QIGTGNFSRVFKVLKRIDG-CMYAVKHSTKQLHQD
Nicotiana tabacum (2)            HEIE-------------------------------QIGTGNFSRVFKVLKRIDG-CMYAVKHSTKQLHQD
Nicotiana tomentosiformis        HEIE-------------------------------QIGTGNFSRVFKVLKRIDG-CMYAVKHSTKQLHQD
Nicotiana sylvestris             HEIE-------------------------------QIGTGNFSRVFKVLKRIDG-CMYAVKHSTKQLHQD
Solanum lycopersicum             HEIE-------------------------------QIGSGNFSRVFKVFKRIDG-CMYAVKHSTKQLHQD
Brachypodium distachyon          HEIEVCSEQILLMCSCSGLTFTSCGDFGHEQSLFQQIGRGNFSLVFKVLRRIEG-CLYAVKRSIKELHSD
Triticum dicoccoides             HEIE-------------------------------QIGYGNFSVVFKVLRRIEG-CLYAVKRSIKQLHND
Oryza sativa                     HEIE-------------------------------QIGRGNFSVVFKVLKRIDG-CLYAVKRSIRQLHND
Zea mays                         HEIE-------------------------------KIGYGNFSVVFKVLNRIDG-CLYAVKRSIKQLHND
Sorghum bicolor                  HEIE-------------------------------KIGYGNFSVVFKVLNRIDG-CLYAVKRSIKQLHND
Daucus carota                    HEIE-------------------------------QIGNGNFSRVFKALKRIDG-CMYAVKHSTRQLHLD
Helianthus annuus                HEIE-------------------------------QIGTGNFSCVFKVLKRIDG-CMYAVKRSTRKLHQD
Cynara cardunculus               HEIE-------------------------------QIGTGNFSRVFKVLKRIDG-CMYAVKCSTRKLYLD
Populus trichocarpa              HEIQ-------------------------------QIGTGNFSCVFKVLKRIDG-CLYAVKHSTRQLHQD
Malus domestica                  HEIE-------------------------------QIGSGYFSHVFKVLNRIDG-CLYAVKRSMKQLHQD
Prunus persica                   HEIE-------------------------------QIGSGYFSHVFKALNRIDG-CLYAVKLSMKQLHQD
Quercus suber                    QEIE-------------------------------KIGTGNFSQVFKVLKRIDG-CLYAVKHSTRQLHLD
Juglans regia                    HEIE-------------------------------QIGAGNFSRVFKVLRRIDG-CLYAVKHSTRQLHQD
Vitis vinifera                   HEIE-------------------------------QIGDGNFSRVFKVLKRIDG-CMYAVKHSTRPLHQD
Citrus sinensis                  HEIE-------------------------------QIGAGNFSHVFKVLKRIDG-CFYAVKHSKRQLHHD
Eucalyptus grandis               HEIE-------------------------------QIGAGNFSCVLKVLKRIDG-CLYAVKQSIRPLYQD
Gossypium hirsutum               HEIE-------------------------------QIGTGHFSRVFKVLKRIDG-CLYAVKHSTRPLHQE
Theobroma cacao                  HEIE-------------------------------QIGTGNFSRVFKVLKRIDG-CLYAVKHTTRQLHQD
Hevea brasiliensis               HEMQ-------------------------------QIGTGNFSRVFKVLKRIDG-CLYAVKHSTRQLHQD
Selaginella moellendorffii       HQIK-------------------------------EIGRGDFSYVYKVLKRLDG-CLYAVKHSNRRLLNE
Physcomitrium patens             HEIR-------------------------------EIGHGNFSRVFKVLKRIDG-CMYAIKRSRHSLRST
Chlamydomonas reinhardtii        HQEA-------------------------------ELGHGNFSKVYKAVHRLTG-IAYAVKTNKVPITTL
Schizosaccharomyces pombe        RNVT-------------------------------LLGSGEFSEVFQVEDPVEKTLKYAVKKLK-VKFSG
Saccharomyces cerevisiae S288C   TNVH-------------------------------SIGKGQFSTVYQVT-FAQTNKKYAIKAIKPNKYNS
Drosophila melanogaster          MQVN-------------------------------VIGVGEFGVVFQCVNRLDG-CIYAIKKSKKPVAGS
Danio rerio                      HELE-------------------------------KIGSGQFGSVFKCVKRLDG-CIYAIKRSKKPLAGS
Mus musculus                     HELE-------------------------------KIGSGEFGSVFKCVKRLDG-CIYAIKRSKKPLAGS
Homo sapiens                     HELE-------------------------------KIGSGEFGSVFKCVKRLDG-CIYAIKRSKKPLAGS
```

G-loop ATP-Bs

```
                                          150       160       170       180       190       200       210
                                 ....|....|....|....|....|....|....|....|....|....|....|....|....|....|
Medicago truncatula              TER----RKALMEVQALAALGY--HENIVGYYTSWFE----NEQLYIQMEICD----HSLSVN---KGSE
Trifolium pratense               TER----RKALMEVQALAALGS--HENIVGYYTSWFE----NEQLYIQMEICD----HSLSVK---KGSE
Pisum sativum                    TER----KKALMEVQALAALGS--HENIVGYYSSWLE----SEQLYIQMEICD----HSLFKF---KGSE
Vicia faba                       TER----KKALMEVQSLAALGS--HENIVGYYSSWFE----SEQLYIQMEICD----HSLSKY---KGSE
Cicer arietinum                  TER----RKALMEVQALAALGS--HENIVGYYSSWFE----NEQLYIQMELCD----HSLSIN---KGYE
Lotus japonicus                  TER----RKALMEVQALAAMGS--HENIVGYYSSWFE----NEHLYIQMELCD----HSLSIN---KCSA
Lupinus angustifolius            TER----KKALMEVQSLAALGS--NENIVGYYSSWFE----NEQLYIQMELCD----HSLSIE---KCSA
Glycine max (Chr3)               TER----TKALMEVQALAALGL--HENIVGYYSSWFE----NEQLYIQMELCD----HSLSIR---KYSA
Glycine max (Chr13)              TER----EKALMEVQALAAIGS--HENIVGYYSSWFE----NEQLYIQMELCD----HSLSIK---NCPS
Phaseolus vulgaris               TER----TKALMEVQALASLGL--HKNIVGYYSSWFE----NEHLYIQMELCD----HSLSIR---KFSA
Vigna unguiculata (Chr1)         TER----TKAMMEVQALAALGL--HQNIVGYYSSWFE----NEQLYIQTELCD----HSLSIR---KCPE
Vigna unguiculata (Chr6.1)       PER----LKAFNEVQSLAAIGR--HKNIVTYYYSWIE----VEQMYTQMEFCD----HNLSYE---NCPI
Vigna unguiculata (Chr6.2)       SE-----WKAYDEVQSLAAIGF--QENIRGYYSSWIE----NKIHYIQMELCD----HSLSMK---SFPA
Arachis hypogaea (Chr16)         TER----RKALMEVQALAALGS--HANIVGYYSSWFE----NEQLYIQMELCD----HSLSIN---KCSS
Arachis hypogaea (Chr6)          TER----RKALMEVQALAALGS--HANIVGYYSSWFE----NEQLYIQMELCD----HSLSIN---KCSS
Cajanus cajan                    TER----TKALMEVQALAALGL--HENIVGYYTSWFE----NEQLYIQMELCD----HSLSIR---KCSQ
Prosopis alba                    TER----RKALMEVQALAALGS--HENIVGYYSSWFE----NERLYIQMELCD----HSLSIS---KANT
Arabidopsis thaliana             SER----RKAMMEVQALAALGF--HENIVGYYSSWFE----NEQLYIQLELCD----HSLSALP-KKSSL
Brassica napus                   SER----CKAMMEVQALAALGF--HENVVGYYNSWFE----NEQLYIQLELCD----HSLS----KKSSL
Nicotiana tabacum (1)            TDR----RKALMEVQALAALGP--HENVVGYYSSWFE----NEHLYIQMELCD----HSLSN---KKYSK
Nicotiana tabacum (2)            TDR----RKALMEVQALAALGP--HENVVGYYSSWFE----NEHLYIQMELCD----HSLSN---KKDSK
Nicotiana tomentosiformis        TDR----RKALMEVQALAALGP--HENVVGYYSSWFE----NEHLYIQMELCD----HSLSN---KKYSK
Nicotiana sylvestris             TDR----RKALMEVQALAALGP--HENVVGYYSSWFE----NEHLYIQMELCD----HSLSN---KKDSK
Solanum lycopersicum             TDR----RQALMEVQALAALGP--HENVVGYYSSWFE----NEHLYIQMELCD----HSLSN---KKYCK
Brachypodium distachyon          RDR----RLALKEVQTLVALGN--HENIVGYFTSWFE----TEKLYIQMELCD----RCLSMN----GNP
Triticum dicoccoides             MDR----RQALKEVHTLVALGN--HENIVGYFTSWFE----TEKLYIQMELCD----RSLSVN----GDK
Oryza sativa                     RER----RQAVKEVQALAALGC--HENIVGYFTSWFE----NKQLFIQMELCD----RCLSMD----RNQ
Zea mays                         MER----RQAVKEVQAMAALGS--HENIVRYFTSWFE----NEQLYIQMELCD----RCLSMN----RNQ
Sorghum bicolor                  MER----RQAVKEVQAMAALGS--HENIVRYFTSWFE----NEQLYIQMELCD----HCLSMN----RNQ
Daucus carota                    MER----RKALMEVQALAALGS--HENIVGYYTSWFE----NEKLYIQLELCE----RSLAI---NGKSK
Helianthus annuus                TER----QKALMEVQALAALGY--HDNIVGYNTSWFE----NEQLYIQMELCD----HNISI---NHASK
Cynara cardunculus               TER----QKALMEVQALAALGY--HDNIVGYNTSWFE----NEQLYIQMELCD----HSLSI---NHSSR
Populus trichocarpa              AER----RKALMEVQALAALGY--HENIVGYYSSWFE----NEQLYIQMELCD----GSLSI---NRSSE
Malus domestica                  TER----RKAMMEVQSLAALGS--HENIVGYYSSWFE----NEQLHIQMELCD----HSLSI---NKFCR
Prunus persica                   TER----RKALMEVQSLAALGS--HENIVGYYSSWFE----NEQLYIQMELCD----HSLSI---YKFSR
Quercus suber                    TER----RKALMEVQSLAALGS--HENIVGYYSSWFE----NEQLYIQMELCD----HSLSM---SRTSQ
Juglans regia                    TER----RKALMEVQALAAVGS--HENIVGYYSSWFE----NEQLYIQMELCD----HSLSM---TKSSQ
Vitis vinifera                   TER----RKALMEVQALAVLGS--HENIVGYYTSWFE----NEKLYIQMELCD----HSLSI---KRSSH
```

```
Citrus sinensis                 AER----RKALLEVQALAALGS--HENIVGYYSSWFE----NEQLYIQMELCD----HSLSD---KKASQ
Eucalyptus grandis              WER----RKALMEVQSLAALGS--HENIVGYYSSWFE----NEHLYIQMELCD----YSLSS---QKSTR
Gossypium hirsutum              TER----RKALMEVQALAALGS--HENIVGYYSSWFE----NEQLYIQMELCD----YSLSR---KSSTQ
Theobroma cacao                 TER----RKALMEVQALAALGS--HENIVGYYSSWFE----NEQLYIQMELCD----YSLSR---NTSLQ
Hevea brasiliensis              AEK----RKALMEVQALAALGC--HENIVGYYSSWFE----NEQLYIQMELCD----CSLSI---NKSSK
Selaginella moellendorffii      GDR----RKALREVQALSCLGY--HENVVRYFSSWFE----NDFLYIQMELCE----TNLRDE---SLAW
Physcomitrium patens            VER----RQALTEVQALAAVGF--HENVVRYYTAWFE----DEYCYIQTELCE----SNLTQLR-DADPN
Chlamydomonas reinhardtii       QAR----NMWLNEMQALAAVQP--HPNIVGLYDTWFEPGGDAEQAYIKQELCG----ESLRDM--FKRRV
Schizosaccharomyces pombe       PK---ERNRLLQEVS--IQRALKGHDHIVELMDSWEH----GGFLYMQVELCENGSLDRFLEEQ--GQLS
Saccharomyces cerevisiae S288C  LKRILLEIKILNEVTNQITMDQEGKEYIIDYISSWKF----QNSYYIMTELCENGNLDGFLQEQVIAKKK
Drosophila melanogaster         SFE----KRALNEVWAHAVLGK--HDNVVRYYSAWAE----DDHMLIQNEFCDGGSLHARIQD------H
Danio rerio                     VDE----QNALREVYAHAVLGQ--HPHVVRYYSAWAE----DDHMLIQNEYCNGGTLSDVIAEN-NRRMH
Mus musculus                    VDE----QNALREVYAHAVLGQ--HPHVVRYFSAWAE----DDHMLIQNEYCNGGSLADAISEN-YRVMS
Homo sapiens                    VDE----QNALREVYAHAVLGQ--HSHVVRYFSAWAE----DDHMLIQNEYCNGGSLADAISEN-YRIMS
```

ATP-Bs

```
                                         220       230       240       250       260       270       280
                                ....|....|....|....|....|....|....|....|....|....|....|....|....|....|
Medicago truncatula             LLAEGQVLEVLYQVANALRFIHDKG-IAHLDLKPDNIYVKN--------------------G--------
Trifolium pratense              FLAEGQVLKAFYQVANALRFMHEKG-IAHLDVKPDNIYVKN--------------------G--------
Pisum sativum                   LLSEGQLLEVLYQVANALRFIHEKG-IVHLDVKPDNIYVKN--------------------G--------
Vicia faba                      LLSEGQLLEVLYQIANALRFIHEKG-IVHLDVKPDNIYVKN--------------------G--------
Cicer arietinum                 LLAEGQALEVLYQVANALQFIHEKG-VAHLDVKPDNIYVKN--------------------S--------
Lotus japonicus                 LLTEGQVLDALYQVANALRFIHEKG-IAHLDVKPDNIYIKN--------------------G--------
Lupinus angustifolius           LFTEGQALEALYQVANALRFIHEKG-IAHLDVKPDNIYVKN--------------------G--------
Glycine max (Chr3)              LFTEGQVLDALFQVANALQFIHEKG-IAHLDVKPDNIYVKN--------------------G--------
Glycine max (Chr13)             LVTAGHEIEALYQVASALQFIHKKG-IAHLDVKPENIYVKN--------------------G--------
Phaseolus vulgaris              AFSEAQVLDALFQVANALQFIHAKG-MAHLDVKPDNIYVKN--------------------G--------
Vigna unguiculata (Chr1)        AFTEAQVLDALFQIANALRFIHEKG-MAHLDVKPNNIYVKS--------------------G--------
Vigna unguiculata (Chr6.1)      LLTERHELQVLYQVSSALQFIHEKG-IAHLHVKPQNIYVKD--------------------G--------
Vigna unguiculata (Chr6.2)      SLSQRYEIEVLYQISSALQFIHGKG-IAHLDVKPENIYVKS--------------------G--------
Arachis hypogaea (Chr16)        SLTEGQVLEALYQIANALRFIHEKG-IAHLDVKPDNIYVKN--------------------G--------
Arachis hypogaea (Chr6)         SFTEGQVLEALYQIANALRFIHEKG-IAHLDVKPDNIYVKN--------------------G--------
Cajanus cajan                   LFTEGQVLDALFQVAKALRFIHEKG-IAHLDVKPDNIYVKN--------------------G--------
Prosopis alba                   MFTEGQVLEALHQVAKALLFIHERG-IAHLDVKPDNIYVKN--------------------G--------
Arabidopsis thaliana            KVSEREILVIMHQIAKALHFVHEKG-IAHLDVKPDNIYIKN--------------------G--------
Brassica napus                  RISEREILVIMHQIAKALQFVHEKG-IAHLDVKPDNIYIKN--------------------G--------
Nicotiana tabacum (1)           LSSEVAVLEAMYQVAKALQYIHQRG-VAHLDVKPDNIYVKS--------------------G--------
Nicotiana tabacum (2)           LFSEVEVLEAMYQVAKALQYIHQRG-VAHLDVKPDNIYVKS--------------------G--------
Nicotiana tomentosiformis       LSSEVAVLEAMYQVAKALQYIHQRG-VAHLDVKPDNIYVKS--------------------G--------
Nicotiana sylvestris            LFSEVEVLEAMYQVAKALQYIHQRG-VAHLDVKPDNIYVKS--------------------G--------
Solanum lycopersicum            LFSEVEVLEAMYQVANALQYIHQRG-VAHLDVKPDNIYVKN--------------------G--------
Brachypodium distachyon         LLKHEEALELLYQVSKGLDFIHGRG-IAHLDVKPDNIYVKN--------------------G--------
Triticum dicoccoides            PLEFGDALELLYQICKGLDFIHGRG-VAHLDVKPDNIYVRN--------------------G--------
Oryza sativa                    PLKCGEALELLYQICKGLDFIHERG-IAHLDVKPDNIYVRN--------------------G--------
Zea mays                        PVKRGEALELLYQICKGLDFMHERG-IAHLDVKPDNIYVRN--------------------G--------
Sorghum bicolor                 PVKHGEAMELLYQICKGLDFMHERG-IAHLDVKPDNIYVRN--------------------G--------
Daucus carota                   LYTEGEVLEAMHQIAKALRYIHERG-VAHLDVKPENIYVKN--------------------G--------
Helianthus annuus               ICSEGEVLEAMHQIAKALQFIHEKG-IVHLDVKPENIYVKN--------------------G--------
Cynara cardunculus              LCLEGEVLEAMHQIAKALQFIHEKG-IVHLDVKPENIYVKN--------------------G--------
Populus trichocarpa             SITEGEALQVLLQIAKALKFIHERG-IAHLDVKPDNIYFKN--------------------G--------
Malus domestica                 PFKEGEILEALYQISKALQLIHGRG-IAHLDVKPDNIYVKN--------------------G--------
Prunus persica                  TFTEGEILEVLFQIAKALQFIHKKG-IAHLDVKPDNIYVKN--------------------G--------
Quercus suber                   LFTEAEVLQALHQIAKALQFIHGRG-IAHLDVKPDNIYVKN--------------------G--------
Juglans regia                   LLAEQEVLLALYQVAKALQFIHERG-IAHLDVKPDNIYVKN--------------------G--------
Vitis vinifera                  LFTEGEVLEAMHQIAKALQFTHEKG-IAHLDVKPDNIYVKD--------------------G--------
Citrus sinensis                 FFAEGEVLKALHQIAQALQFIHEKG-IAHLDVKPDNIYVKN--------------------G--------
Eucalyptus grandis              -LTEWDALTALYQLAKALLFMHERG-IAHLDVKPDNIYVKN--------------------G--------
Gossypium hirsutum              LFTEGEFLRAMHQMAKALQFIHEKG-IVHLDVKPDNVYVKN--------------------G--------
Theobroma cacao                 LFTEAEVLRALLQIAKALQCIHERG-IAHLDVKPDNIYVKN--------------------G--------
Hevea brasiliensis              SITEDKVLEVLHQIAKALHFIHGCG-IAHLDVKPDNIYVKN--------------------G--------
Selaginella moellendorffii      TFTEKKLTEAMFQLLNALKHLHSHG-LAHLDVKPDNIYIRK--------------------G--------
Physcomitrium patens            VSKETSLLEIMRQMASALSAIHSKG-LVHLDVKPDNIYVLN--------------------G--------
Chlamydomonas reinhardtii       QFKEVEVLEILRQMASALKRIHDLG-MVHLDIKPDNIYLAAPSPADLALRAASGANASSSAGSSSGGSS-
Schizosaccharomyces pombe       RLDEFRVWKILVEVALGLQFIHHKN-YVHLDLKPANVMITFEG---------------------------
Saccharomyces cerevisiae S288C  RLEDWRIWKIIVELSLALRFIHDSCHIVHLDLKPANVMITFEG---------------------------
Drosophila melanogaster         CLGEAELKIVLMHVIEGLRYIHSND-LVHMDLKPENIFSTMNPNAHKLVEVQPQQT-KDDDGMDSVYEEL
Danio rerio                     FLSELELKDLLLQVSRGLKYIHSTA-LVHMDIKPSNIFISRK--PAASVEEFE----DEEDGPT------
Mus musculus                    YLTEVELKDLLLQVGRGLRYIHSMS-LVHMDIKPSNIFISRTSIPNAVSEEG-----DEDDWIS------
Homo sapiens                    YFKEAELKDLLLQVGRGLRYIHSMS-LVHMDIKPSNIFISRTSIPNAASEEG-----DEDD AS------
```

Catalytic segment

```
                                         290       300       310       320       330       340       350
                                ....|....|....|....|....|....|....|....|....|....|....|....|....|....|
Medicago truncatula             -------VYKLGDFGCATLTDSS-LP-IEEGDARYMPQEILNEN----YDHLDKVDIFSLGVSIYQLVRK
```

```
Trifolium pratense              -------VYKLGDFGCATLIDSS-LP-IEEGDARYMPQEILNDN----YDHLDKVDIFSLGASIYELVRK
Pisum sativum                   -------VYKLGDFGCATLIDNS-LP-IEEGDARYMPQEILNEK----YDHLDKVDIFSLGASIYELVRK
Vicia faba                      -------VYKLGDFGCATLIDNS-LP-IEEGDARYMPQEILNEK----YDHLDKVDIFSLGASIYELVRK
Cicer arietinum                 -------VYKLGDFGCATLIDNS-LP-IEEGDARYMPQEVLNEN----YDHLDKVDIFSLGASIYELIRR
Lotus japonicus                 -------VYKLGDFGCATLLDTS-LP-IEEGDARYMPQEILNEN----YDHLDKVDIFSLGASIYELIRR
Lupinus angustifolius           -------VYKLGDFGCATLLDNS-LP-IEEGDARYMPQEILNEN----YDHLDKVDIFSLGASIYELIRR
Glycine max (Chr3)              -------VYKLGDFGCATLLDSS-LP-IEEGDARYMPQEILNEN----YDHLDKVDIFSLGASIYELIRR
Glycine max (Chr13)             -------VYKLGDFGCATLLDNS-LP-IEEGDAHYMPQEILNEN----YDHLDKVDIFSLGASMFELISS
Phaseolus vulgaris              -------VYKLGDFGCATLLDSS-LP-IEEGDARYMPQEILNEN----YDNLDKVDIFSLGASIYELIRR
Vigna unguiculata (Chr1)        -------VYKLGDFGCATLLDGS-LP-IEEGDARYMPQEILNEN----YDHLDKVDIFSLGASIYELIRR
Vigna unguiculata (Chr6.1)      -------IYKLGGFRCATLLDNS-LH-VEAGDAQYMPPEILNEN----YDHLGKADIFSLGASMFDIIQR
Vigna unguiculata (Chr6.2)      -------VYKLGGFGYATLLDDN-VL-VEAGDAQYMPLEILNEN----YDHLDKADIFSLGASMFDIIRR
Arachis hypogaea (Chr16)        -------VYKLGDFGCATLLDNS-LL-VEEGDARYMPQEILNDN----YDHLDKVDIFSLGASVYEFIRR
Arachis hypogaea (Chr6)         -------VYKLGDFGCATLLDNS-LL-VEEGDARYMPQEILNDN----YDHLDKVDIFSLGASVYEFIRR
Cajanus cajan                   -------VYKLGDFGCATLIDSS-LP-VEEGDARYMPQEILNEN----YDHLDKVDIYSLGASIYELIRG
Prosopis alba                   -------VYKLGDFGCATLLDKS-LP-IEEGDARYMPQEILNEN----YDHLDKVDIFSLGASIFELIRG
Arabidopsis thaliana            -------VCKLGDFGCATRLDKS-LP-VEEGDARYMPQEILNED----YEHLDKVDIFSLGVTVYELIKG
Brassica napus                  -------VCKLGDFGCATRLDKS-LP-VEEGDARYMPQEVLNEN----YEHLDKVDIFSLGVTVYELIRG
Nicotiana tabacum (1)           -------VYKLGDFGCATLLDKS-QP-IEEGDARYMPQEILNEN----YDHLDKVDVFSLGAAIYELIRG
Nicotiana tabacum (2)           -------VYKLGDFGCATLLDKS-QP-IEEGDARYMPQEILNEN----YDHLDKVDVFSLGAAIYELIRG
Nicotiana tomentosiformis       -------VYKLGDFGCATLLDKS-QP-IEEGDARYMPQEILNEN----YDHLDKVDVFSLGAAIYELIRG
Nicotiana sylvestris            -------VYKLGDFGCATLLDKS-QP-IEEGDARYMPQEILNEN----YDHLDKVDVFSLGAAIYELIRG
Solanum lycopersicum            -------VYKLGDFGCATLLDKS-QP-IEEGDARYMPQEILNEN----YDHLDKVDIFSLGAAIYELIRG
Brachypodium distachyon         -------VYKLGDFGCATLIDRS-LP-IEEGDARYMPPEMLNDE----FEHLDKVDIFSLGATVYELIRG
Triticum dicoccoides            -------IYKLGDFGCATLIDRS-LA-IEEGDSRYMPPEMLNEK----HEHLDKVDIFSLGAAVYELIRG
Oryza sativa                    -------VYKLGDFGCATLIDRS-LA-IEDGDSRYMPPEMLNDK----YEHLDKVDIFSLGAAIYELIRG
Zea mays                        -------IYKLGDFGCATLVNRS-LA-IEDGDSRYMPPEMLNDK----YEHLDKVDIFSLGAAVYELIRG
Sorghum bicolor                 -------IYKLGDFGCATLINRS-LA-IEDGDSRYMPPEMLNDK----YEHLDKVDIFSLGAALYELIRG
Daucus carota                   -------LYKLGDFGCATLLDAS-LP-IEEGDARYMPQEILNEN----YDHLDKVDIFSLGATIYELVKG
Helianthus annuus               -------VYKLGDFGCASLLDGS-LP-IDEGDARYMPQEILNDD----YDHLDKVDIFSLGATIYEIIRG
Cynara cardunculus              -------VYKLGDFGCASLLDGS-LA-IEEGDARYMPQEILNDN----YDHLDKVDIFSLGATIYELIRG
Populus trichocarpa             -------VYKLGDFGCATLLDQS-LP-VEEGDARYMPQEILNEN----YNYLDKVDIFSLGAAIYELIRG
Malus domestica                 -------VYKLGDFGCATLLDNS-LP-IEEGDARYMPQEILNEK----YDDLDKADIFSLGVAVYELIRG
Prunus persica                  -------VYKLGDFGCATLLDKS-LP-IEEGDARYMPQEILNEK----YDDLDKADIFSLGVAIYELIRG
Quercus suber                   -------VYKLGDFGCATLTDKS-LP-IEEGDARYMPQEILNEK----YDHLDKVDIFSLGASIYELIRG
Juglans regia                   -------VYKLGDFGCATLTDKS-LP-IEEGDARYMPQEILNEK----YDYLDKVDIFSLGAAIYELIRG
Vitis vinifera                  -------VYKLGDFGCATLLDRS-LP-IDEGDARYMPQEILNDN----YDHLDKVDMFSLGAAIYELIRG
Citrus sinensis                 -------VYKLGDFGRATLLNKS-LP-IEEGDARYMPQEILNED----YDHLDKVDIFSLGATMYELIKG
Eucalyptus grandis              -------VYKLGDFGCATLLNSS-LP-VEEGDARYMPQEILNDN----FDHLDKVDIFSMGASMYELIRG
Gossypium hirsutum              -------VYKLGDFGCATLLNKN-LP-VEEGDARYMPQEILNEN----YDHLDKVDIFSLGVTIYELIRG
Theobroma cacao                 -------VYKLGDFGCATLLNKS-LP-IEEGDARYMPQEILNEN----YDHLDKVDVFSLGVTIYELIRG
Hevea brasiliensis              -------VYKLGDFGCATLLNQS-LP-IEEGDARYMPQEILNEN----YNHLDKVDIFSLGAAIYELIRG
Selaginella moellendorffii      -------VYKIGDFGLACRIDGA-IS-IEDGDSRYLPMELINDD----HSHLDKADMFSLGATFYELARR
Physcomitrium patens            -------VYKLGDFGRAVRADGS-ME-IEEGDARYMPLEILNDD----HSQLTKVDMFSLGATIYELARG
Chlamydomonas reinhardtii       ----AAPVYKLGDFGLAMLPGGQ-RAGTSEGDVKYLAPEALKSRDFLTSGLADRLDVFALGASVYELLRG
Schizosaccharomyces pombe       -------TLKIGDFGMASVWPVP-RGMEREGDCEYIAPEVLANH-----LYDKPADIFSLGITVFEAAAN
Saccharomyces cerevisiae S288C  -------NLKLGDFGMATHLPLEDKSFENEGDREYIAPEIISDC-----TYDYKADIFSLGLMIVEIAAN
Drosophila melanogaster         RHSENLVTYKIGDLGHVTSVK---EPYVEEGDCRYLPKEILHED----YSNLFKADIFSLGITLFEAAGG
Danio rerio                     ----TNVVYKIGDLGHVTTVT---NPQVEEGDSRFLANEVLQED----YSNLKKADIFALALTVVSASGA
Mus musculus                    ----NKVMFKIGDLGHVTRIS---SPQVEEGDSRFLANEVLQEN----YSHLPKADIFALALTVVCAAGA
Homo sapiens                    ----NKVMFKIGDLGHVTRIS---SPQVEEGDSRFLANEVLQEN----YTHLPKADIFALALTVVCAAGA
```

Activation segment

```
                                     360       370       380       390       400       410       420
                                ....|....|....|....|....|....|....|....|....|....|....|....|....|
Medicago truncatula             --SPMPESGCHFLNLKEGKLPLLP-GHSLQ----------------------FQNLLKAMIDPDPVKRP
Trifolium pratense              --SPLPESGGHFLNLKEGKLPLLP-GHSLQ----------------------FQNLLKAMIDPDPVKRP
Pisum sativum                   --SPLPESRCHFSNLKEGKLSLLP-SNTMQ----------------------FQNLLKAMIDRDPVKRP
Vicia faba                      --SPLPESRCHFSNLKEGKLSLLP-SNTMQ----------------------FQNLLKAMIDRDPVKRP
Cicer arietinum                 --LPLPESGSHFLNLKEGKLPLLP-GHSLQ----------------------FQNLLKAMMDPDPVKRP
Lotus japonicus                 --LPLPDSGCNFLNLKGGKLPLLP-GHSLQ----------------------LQNLLKVMMDPDPVKRP
Lupinus angustifolius           --LPLPDSGCHFLNLKEGKLRLLP-GHSLQ----------------------FQNLLKVMMDPDPVKRP
Glycine max (Chr3)              --LPLPESGCQFFNLKEGKFPLLP-GHSLQ----------------------LQNLLKVMMDPDPVKRP
Glycine max (Chr13)             SCLPEPESGIQFFNPKEGKVPFLP-GVSVQ----------------------FQNLLTVMMDPDPVKRP
Phaseolus vulgaris              --LPLPESGCQFFNLKEGKFPLLP-GHSLH----------------------LQNLLKVMMDPDPVKRP
Vigna unguiculata (Chr1)        --LPLPESGCQYFNLKEGKFPLLP-GHSLH----------------------IQNLLKVMMDPDPVKRP
Vigna unguiculata (Chr6.1)      --SRLPKAETEFSKLKEGKVPHLP-GVTIQ----------------------FQNLLKVMMDPDPLKRP
Vigna unguiculata (Chr6.2)      --SRLPRAETEFSKLKEGKVPHLP-GVTVQ----------------------FQNLLKVMMDPDPLKRP
Arachis hypogaea (Chr16)        --LPLPESGCQFFNLKEGKLPLLP-GHSLQ----------------------FQNLLKAMMDPDPVKRP
Arachis hypogaea (Chr6)         --LPLPESGSQFFNLKEGKLPLLP-GHSLQ----------------------FQNLLKAMMDPDPVKRP
Cajanus cajan                   --SLLPESGDQFLNLKEGKLTLLP-GHSLQ----------------------LQNLLKVMMDPDPVKRP
Prosopis alba                   --SPLPESGCKFFNLKEGRLPLLP-GHSLQ----------------------FQNLLKVMMDPDPVKRP
Arabidopsis thaliana            --SPLTESRNQSLNIKEGKLPLLP-GHSLQ----------------------LQQLLKTMMDRDPKRRP
Brassica napus                  --SPLTESRNKSLNIKQGKLPLLP-GHSLQ----------------------LQQLLKTMMDRDPSRRP
Nicotiana tabacum (1)           --SPLPESGPHFLNLREGKLPLLP-GHSLQ----------------------FQNLLKVMMDPDPTRRP
Nicotiana tabacum (2)           --SPLPESGPHFLNLREGKLPLLP-GHSLQ----------------------FQNLLKVMMDPDPTRRP
```

```
Nicotiana tomentosiformis          --SPLPESGPHFLNLREGKLPLLP-GHSLQ-----------------------FQNLLKVMMDPDPTRRP
Nicotiana sylvestris               --SPLPESGPHFLNLREGKLPLLP-GHSLQ-----------------------FQNLLKVMMDPDPTRRP
Solanum lycopersicum               --SSLPESGPHFLNLREGKLPLLP-GHSLQ-----------------------FQNLLKAMMDPDPTRRP
Brachypodium distachyon            --TPLPLSGPQFTSLREGKFALLP-GRPIQ-----------------------FQNLIKLMMDPDPTRRP
Triticum dicoccoides               --TPLPVSGHQFASLREGKISLLP-GHPMQ-----------------------FQSLIKSMMDPDPVRRP
Oryza sativa                       --TQLPDSGPQFTSLREGKIALLP-GCPMQ-----------------------FQSLIKSMMDPDPVRRP
Zea mays                           --TPLPESGSHFTSIREGKIALLP-GCPMQ-----------------------FQSLIKSMMDPDPVRRP
Sorghum bicolor                    --TPLPESGPHFTSIREGKIALLP-GCPMQ-----------------------FQSLIKSMMDPNPVRRP
Daucus carota                      --STLPESGPHFLHLREGKLPLLP-GHSVQ-----------------------FQSLLKAMMDPDPMRRP
Helianthus annuus                  --STLPDSGPYFQNLREGKLPLLP-GHSVQ-----------------------FQNILKAMLDPDPVRRP
Cynara cardunculus                 --STLPESGPYFQNLREGKLPLLP-GHSVQ-----------------------FQNVLKAMLDPDPVRRP
Populus trichocarpa                --STLPQSGSHLFNLREGKLPLLP-GHSLQ-----------------------LQNLLKAMVCPDPIRRP
Malus domestica                    --SPLPESGPQILNLREGKLPLLP-GHSLQ-----------------------FQNLLKFMLDPNPVWRP
Prunus persica                     --LPLPESGPQILNLREGKLPLLP-GHSLQ-----------------------FQNLLKVMLDPNPVWRP
Quercus suber                      --SPLPEAGSQFL--KEGKLALLP-GHSLQ-----------------------FQNLLKVMVDPDPVRRP
Juglans regia                      --SPLPESRSQFLNLKEGKLPLLP-GHSLQ-----------------------FQNLLKAMVDPDPVRRP
Vitis vinifera                     --SHLPESGYQFLNLREGKLPLLP-GYSLQ-----------------------FQNLLKAMLDPDPVQRP
Citrus sinensis                    --SPLSESGPQFLNLRDGKLPLLP-GHSLQ-----------------------FQNLLKVMVDPDPVQRP
Eucalyptus grandis                 --SPLPESGPQFLNLREGKLPLLP-GHSLH-----------------------FQNLLKVMMDPDPTTRP
Gossypium hirsutum                 --SPLPESGPQFL--REGKLPLLP-GYSLQ-----------------------FQNLLKVMVDHDPVRRP
Theobroma cacao                    --SPLQESGRQFL--REGKLPLLP-GHSIQ-----------------------FQNLLKVMVDQDPVRRP
Hevea brasiliensis                 --SPLPKSGSQFFSLREGKLPLLP-GHSLH-----------------------FQNILKVMLDPDPVQRP
Selaginella moellendorffii         --SPLPASGSQYQAIRQGKLALLP-GFSLV-----------------------FQSLIKSLMHPAAKNRP
Physcomitrium patens               --LPLPTSGAQFQSLREGKLSLLP-GYSLT-----------------------LQNIFKALLNPDASARP
Chlamydomonas reinhardtii          --TELPKNGQSYHDIRQGKL-FLP-SASTR-----------------------IINLLKKMMSPDPAQRP
Schizosaccharomyces pombe          --IVLPDNGQSWQKLRSGDLSDAPRLSSTD-NGSSLTSSSRETPANSIIGQGGLDRVVEWMLSPEPRNRP
Saccharomyces cerevisiae S288C     --VVLPDNGNAWHKLRSGDLSDAGRLSSTDIHSESLFSDITKVDTNDLFDFE-RDNISGNSNNAGTSTVH
Drosophila melanogaster            --GPLPKNGPEWHNLRDGKVPILP-SLSRD-----------------------FNELIAQMMHPYPDKRP
Danio rerio                        --EPLPTNGDKWHKIRQGILPHIPQVLSQE-----------------------FLSLLKLMIHPDPTRRP
Mus musculus                       --EPLPRNGEQWHEIRQGRLPRIPQVLSQE-----------------------VTELLRVMIHPDPERRP
Homo sapiens                       --EPLPRNGDQWHEIRQGRLPRIPQVLSQE-----------------------FTELLKVMIHPDPERRP

                                           430
                                   ....|....|.
Medicago truncatula                SARELVENPIF
Trifolium pratense                 SARELVENPIF
Pisum sativum                      SARELVENPIF
Vicia faba                         SARELVENPIF
Cicer arietinum                    SARELVENPIF
Lotus japonicus                    SARELVENPIF
Lupinus angustifolius              SARELVENPIF
Glycine max (Chr3)                 SAKELIENPIF
Glycine max (Chr13)                SATKLLKRVL-
Phaseolus vulgaris                 SAKELVENPIF
Vigna unguiculata (Chr1)           SARELVENPIF
Vigna unguiculata (Chr6.1)         SATEILGNSIF
Vigna unguiculata (Chr6.2)         SATEILGNSIF
Arachis hypogaea (Chr16)           SARELIENPIF
Arachis hypogaea (Chr6)            SARELIENPIF
Cajanus cajan                      SAKELVENPIF
Prosopis alba                      SARELVENTIF
Arabidopsis thaliana               SARELLDHPMF
Brassica napus                     SARELVEHPMF
Nicotiana tabacum (1)              SAKDLVDNPIF
Nicotiana tabacum (2)              YAKDLVDNPIF
Nicotiana tomentosiformis          SAKDLVDNPIF
Nicotiana sylvestris               YAKDLVDNPIF
Solanum lycopersicum               SAKGVVDNPIF
Brachypodium distachyon            SAKEILRHPIF
Triticum dicoccoides               SAKEILRHPIF
Oryza sativa                       SAKEVLRHPIF
Zea mays                           SAKEILRHPSF
Sorghum bicolor                    SAKEILRHPSF
Daucus carota                      SAKEVVENQLF
Helianthus annuus                  SAKELVVNPIF
Cynara cardunculus                 SAKELVVNPIF
Populus trichocarpa                SAKELIENPMF
Malus domestica                    SAKDLVENPIF
Prunus persica                     SAKDLVENPIF
Quercus suber                      SAKELLENPIF
Juglans regia                      SAKELVQNTIF
Vitis vinifera                     SAKELVENPIF
Citrus sinensis                    SAKELVENPIF
Eucalyptus grandis                 SAKELVANPIF
Gossypium hirsutum                 SAKELVENPIF
Theobroma cacao                    SAKELIENPIF
Hevea brasiliensis                 SAKELLENPIF
```

Selaginella moellendorffii	SAAQALKNALF
Physcomitrium patens	SAAELLNHSVF
Chlamydomonas reinhardtii	TADGILRSTLL
Schizosaccharomyces pombe	TIDQILATDEV
Saccharomyces cerevisiae S288C	NNSNINNPNMN
Drosophila melanogaster	TSQSIFSHPIL
Danio rerio	STSELVRHPVL
Mus musculus	SAMELVKHSVL
Homo sapiens	SAMALVKHSVL

Supplementary Figure 2

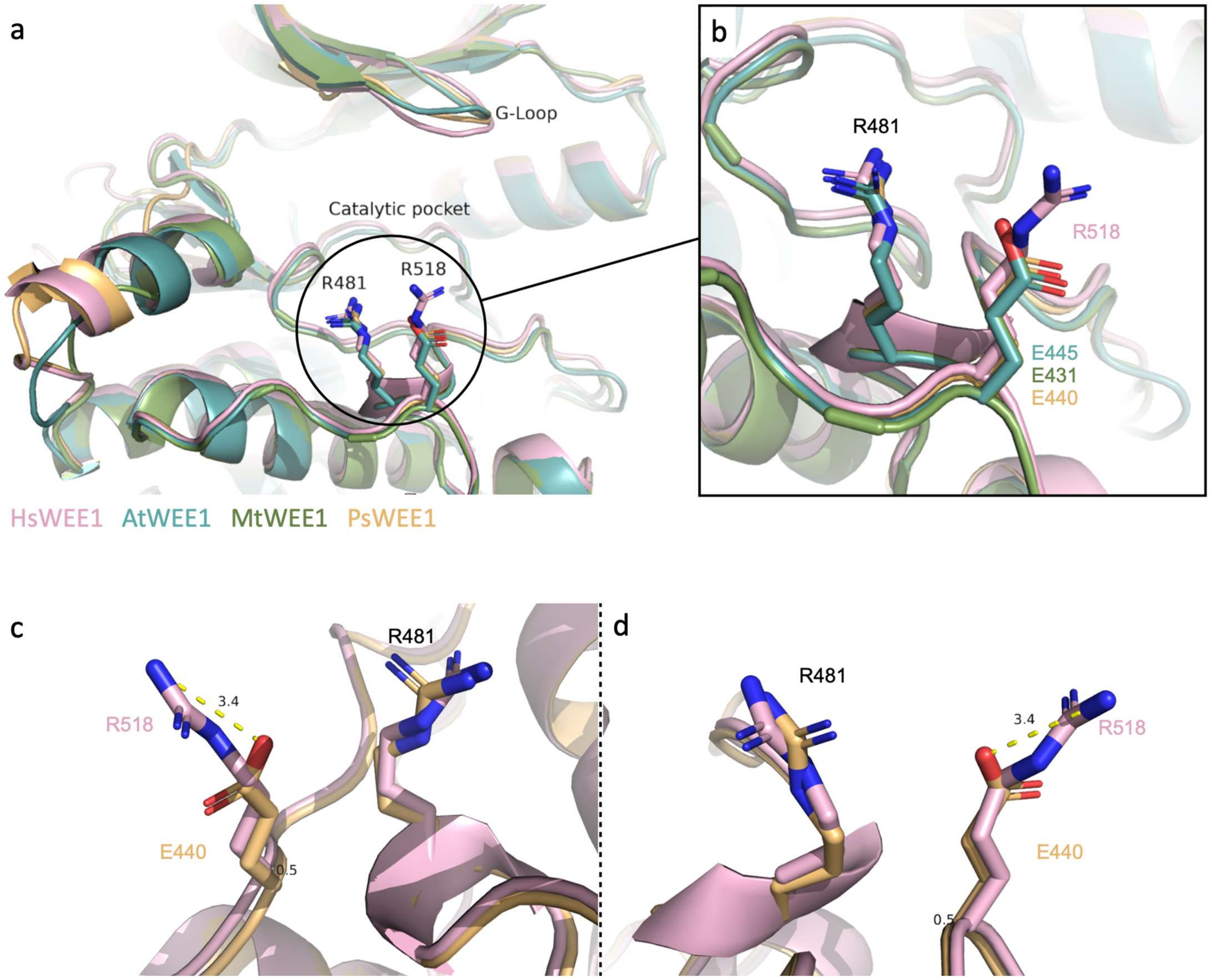

Supplementary Figure 3

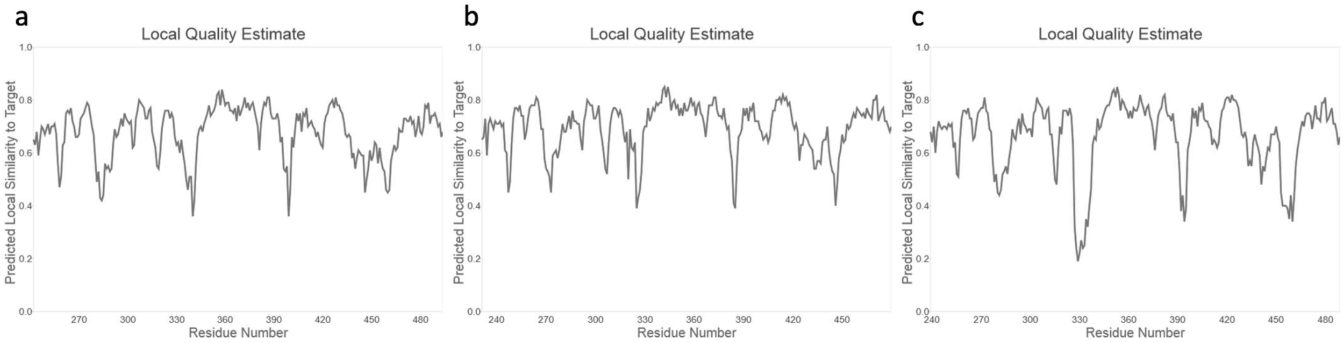

Supplement: Supplementary file 2 — Supplementary Figures. [file 41598_2021_3268_MOESM2_ESM.pdf]
